# Study of Comparative Anatomy

**Published:** 1891-03

**Authors:** A. H. Thompson

**Affiliations:** Topeka, Kan.


### SELECTIONS.

### The Study of Comparative Anatomy, and its Value to Dentists.*

BY A. H. THOMPSON, D.D.S., TOPEKA, KAN.

It is universally conceded by students in all branches of learning, that the comparative method in any study is the only scientific method. It is not enough that one branch be studied alone and exclusively, be that study and investigation ever so minute and thorough; for although thoroughness and minuteness will make depth of learning, it is not enough. There must be breadth of knowledge as well as depth, and to insure breadth, a given branch must be compared with collateral branches. Exclusive attention to one thing will make a deep student, but a narrow mind. It requires frequent excursions into other fields to gather material for the illumination of one's special branch, to make the broad and cultured mind. Specialists and special branches are necessary, of course, and exclusive devotion of the mind to one field is required to bring out all that can be known on even one branch—for human time and strength are limited and one mind can not grasp all knowledge; but that devotion does not exclude everything else, but rather includes a knowledge of those collateral branches which serve to illuminate the special field, for its better understanding.

Especially is this true in regard to the study of man since he has come to be scientifically studied as an animal, in his relations to the rest of the animal kingdom. Before this era, when man abrogated himself a sort of divine superiority over the rest of nature, he would not submit his royal person to comparison with those of other animals. Now, however, as Dr. Holmes has it, "man is studied as the rocks and stars," and the scalpel, the test-tube and the microscope are applied to him as to other objects in nature, which are to be studied and investigated.

Therefore the comparative method in the study of man is now recognized as the only scientific method, and is the only one generally pursued. Not only are his anatomy and physiology illuminated and better understood by comparison with that of other animals, but even his psychology is now being subjected to this method. The old system of psychology, like the old system of biology, will probably go to the wall, and many idols will fall before the irreverent sweep of the comparative method ; but it will bring us nearer a correct understanding of the mind and its faculties.

Prof. Joseph Le Conte ("Evolution and Religious Thoughts") says : "There are two widely distinct views concerning the relation of man to nature ; the one as old as the history of human thought, the other only now urged upon us by modern science. According to the one, man is the counterpart and equivalent of nature ; he alone has, in fact is, an immortal spirit, and therefore belongs to a world of his own. According to the other, man is but a part, a very insignificant part, of nature, and connected in the closest way with all other parts, especially with the animal kingdom. He has no world of his own, or even kingdom of his own, he belongs to the animal kingdom. In that kingdom he has no department of his own, he is a vertebrate. In the department of vertebrates he has no privileged class of his own ; he is a mammal. In the class of mammals he has no titled order of his own ; he is a primate, and shares his primacy with the apes. It is doubtful if he may enjoy the privacy of a family of his own—the Homide—for the structural differences between him and the anthropoid apes are probably not so great as between, for instance, the sheep family and the deer family.

"Now, it is evident that these two standpoints are only views from different points, the psychical and structural. From the psychical point of view it is simply impossible to aggregate the vastness of the gap that separates man from even the highest mammals. From the structural point of view, on the contrary, it is impossible to exaggerate the closeness of the connection. Man's body is identified with all nature in its chemical constituents, with the bodies of all animals in its functions, with all vertebrates, especially mammals, in its structure. Bone for bone, muscle for muscle, ganglion for ganglion, almost nerve-fiber for nerve fiber, his body corresponds with that of the higher mammals. Whether he is derived from lower animals or not, certain it is that his structure, even in its minutest details, is precisely such as it would be if he were thus derived by successive slight modifications.

"Now of these two views the latter has been in recent times earnestly productive in increasing our knowledge. Anatomy has become truly scientific only through comparative anatomy; physiology through comparative physiology; embryology through comparative embryology. Sociology is fast following in the same line, and becoming scientific through comparative sociology. Is not the same true also of psychology? Will not psychology become truly scientific only through comparative psychology, *i. e.*, by the study of the mind of man in relation to the mental life of lower animal?"

But it is to the study of comparative anatomy especially, and its methods, that we wish now to direct attention. This study is attractive and fascinating to those to whom, like specialists in other fields, it elects to open its doors. It is not necessary to defend its beauties now, but rather to point out its value and utility in the study of man, and especially regarding the illumination it bestows upon our knowledge of the jaws and teeth. In this field it has not been utilized as it should be and the especial plea of this paper is for the comparative study of the teeth in our colleges, and that it may receive more attention than it has heretofore. The study will clear up many obscure points in the forms and structure of the teeth of man, and give the student a far better understanding of the principles which govern their evolution and organization. It will, in fact, make odontology scientific.

And first as regards the great principles. The leading principles of comparative study are, the Analogy and Homology of parts and organs. As Prof. Le Conte says (op. cit.): "In biology those organs or parts in different animals are said to be analogous which, however different their origen, have a general similarity of form and especially of function; while those are called homologous which, however different their general appearance and however different their function, yet may be shown to be modifications of originally the same part, but altered for different purposes. The analogous parts when compared look and behave as if they were the same, yet are not; and the homologous parts look and behave very differently but are, in fact, the same part in disguise. For example, the wing of a bird and the wing of a butter-fly are analogous organs and have the same functions—flying. But they are not homologous, for they are not the same organ or part and have certainly never been formed out of the same original organ by modification. But the wing of a bird, the forepaw of a reptile or mammal, the wing of a bat, the arm and hand of man, though so different in form and function, are homologous parts. On close examination they are found to have the same general structure, to be composed essentially the same pieces, although so greatly modified in order to adapt them to different function that the general resemblance is lost. They are homologous but not analogous parts. Their structure is precisely such as it would be if they had all originated from some archetype fore limb by modifications for different purposes. Again, the lungs of a mammal and the gills of a fish are analogous organs, since they have the same function of aeration of the blood. But they are not homologous, they are not built with the same plan, nor could one be derived from the other. But there is an organ in the fish which is homologous with the mammalian lung—the air bladder—which is used by fishes only for flotation. This organ is the beginning of lungs, which by gradual steps is developed into the lungs of the higher air breathing animals." These examples might be multiplied to the extent of all the organs and tissues of the body, but we see clearly that "analogy has reference to general resemblance of form determined by similarity of function, however different the origin of the parts may be. Homology has reference to community of origin, however obscure the path of evolution may be and however diverse may be the organs and their functions now. Common origin completely explains homology by comparison, and by the study of embryology and the comparative method is therefore the scientific method."

It is by homology of structure, not by superficial appearances, that the whole animal kingdom is divided and classified. All animals are grouped in relation to their plan of structure. There are first the two great divisions of the animal kingdom into ver tebrates and invertebrates. The first has a backbone or vertebral column, the other has no backbone, but most of them have the body divided into segments or joints. The general structure in each class is governed and the organs are arranged according to the primary plan. These sub-kingdoms are then divided and sub-divided until division can be carried no further when it reaches species; each division depending on peculiarities of structure and classification depending upon homologies.

Restricting ourselves to the vertebrates, there is to be noticed as a special example of homology, that of the limbs. Compare the fore limb of man with that of the cat, dog, sheep, horse, bat bird, turtle, frog, mole, whale or fish—and what a variety of function is represented, but throughout the series there is homology of the whole and harmony of structure, for there is a similar origin of each one and similar embryology and development. The law of differentiation comes in, in the development and specialization of forms, by which the law of adaptation of structure, while the general plan remained the same. This law of differentiation is the fundamental law of evolution. It first causes the divergencies that take place in the process of the evolution of the cells forming the embryo, by which the typal destiny of the individual is indicated. The cells diverge again to form different tissues, and those to form different organs, to perform different functions. So in the formation of the limbs, the different cast was given to each one as the ruling type indicated, at a certain stage in the development of the individual, and the law operates also in regard to the evolution of all other limbs and organs.

The development of the mammalian head is of special interest to us as dentists, and more particularly the face, jaws and teeth, the brain case has been modified wonderfully, from the lowest to the highest vertebrates, to accommodate the growing brain, until in man it towers over the face, and the jaws become reduced to a more or less rudimentary condition as compared with other animals. The face has some curious analogues in the animal series, especially when comparing its structure as evolved for the accommodation of the sense organs with the sense organs of other forms. For instance, in the vertebrates the sense organs have a definite position in the head and a definite relation to the brain, so that in the mammalia the face and sense organs are analogous and homologous. As compared with lower forms, however, it is a mooted question as to what the jaws really are and what are their homologies? It is known that the jaws are developed from the two upper segments or rings of gill-clefts of fishes and amphibians, but it is not settled whether they are homologous with the limbs or not. They are appendicular structures like the limbs and are, like them, suspended from the vertebral skeleton, but are probably not homologous. It is well known from the developing embryo where the upper pairs of gill-clefs unite to form the upper and lower jaws, but their homology is obscure.

Throughout the vertebraic series, from the fishes and amphibians up through the reptiles, birds and mammals to man, the jaws are homologous and analogous and from origin to function pursue strict relationship and resemblance. They vary somewhat as regards the extra functions performed by them in different animals, but the main purpose is preserved in all. In the herbivorous mammals, for instance, there is extensive masticating area and a loose moveable jaw to allow of free movement of the lower jaw, or mandible, in every direction, for the minute comminution of coarse vegetable food. In the carnivora there is simple vertical movement for the seizing and cutting of flesh, but without lateral movement of the jaws, as the flesh food does not require mastication. In the rodents there is extensive vertical movement to allow of cutting with the strong incisors, and considerable lateral motion to permit of mastication. In the more omnivorous forms there is a combination of these movements without the extremes, and in all forms there is an adaption of means to ends. The study of the mechanism of the jaws in lower forms, their origin, development and function, comparing them step by step with those of man, would throw much light on many questions. For instance, much interesting information regarding the elucidation of the vexing problem of the causes of irregularities of the teeth, could be acquired by the extensive study of the jaws in lower forms and even in the lower races of man, as compared with the higher races. Many resemblances could be worked out which would account for erratic forms of the jaws in man, which we now consider to be abnormal freaks, might only be reversals to former type.

In the field of the study of the teeth, odontography, in relation to and in comparison with those of lower animals, there is a mass of interesting material to be gathered. Every one knows that the teeth in man are more or less rudimentary as compared with other forms, and it is only by comparison with those forms that a proper understanding of them can be attained. The developmental history of a tooth is as interesting as that of a species. The light that is thrown upon our special field by such investigation, is very great, and if we would pursue it would help us to understand many things which are now regarded with childish wonder as mere curiosities or abnormalities, but which are really subject to simple laws, if we would but study and understand them. We are far behind the science of the age in our methods of studying the teeth, and in the quantity and quality of the knowledge of the subject which we know and teach. The mass of the profession know little of the comparative anatomy or its value in our field; and so far as the young men are concerned, the fault lies with the instructors and the system in the colleges. Accounts often appear in the journals of anomalies in the possession of correspondents, which are described as being very wonderful and unique, but which the student of comparative anatomy knows are merely reversals. For instance, a three-rooted, upper biscuspid is a reversal to the anthropoid type, as the apes have that form, their premolars being implanted with three roots. Or again, an additional incisor, a double, it may be, is reported, which is also a reversal (the original number of incisors, being six, two having been suppressed), or one or both laterals may be suppressed entirely, and for several generations in a family. This the student knows is because the lateral incisor is becoming erratic, like the wisdom tooth, in less degree, and like it, is starting on the road to disappearance. The wisdom-tooth is quite often absent in civilized man, because, as is well known, it is on the high road to total suppression in the species, although it is as good and as well set in the lower races of man and the higher apes, as the other molars.

This suggests the subject of additional or extra teeth in the human series, "supernumerary teeth," as they are called. These are usually considered to be freaks and accidents; but the evolutionist knows that they are produced by the workings of the law of atavism, *i. e.*, the reappearance of organs or parts that have, in the course of development, been aborted. Man has lost twelve teeth, *i. e.*, the difference between thirty-two. and forty-four, the normal, typical mammalian number, and supernumerary teeth are the representatives of the number that have been suppressed. Nature, in response to some unknown impulse, makes a spasmodic effort, a spurt of hereditary energy, and reproduces the original dental germs. Additional molars, bicuspids, or incisors, which were the last teeth to disappear from the species, are usually the teeth to reappear. They are, of course, mostly deformed and rudimentary, as the effort to produce teeth of full form is too much for the failing powers. Occasionally a fully formed supernumery tooth is produced, but the instances are rare.

There are occasional resemblances to lower forms which crop out in individual teeth that are highly interesting, and but for the light thrown upon these eccentricities by comparative study, would be considered as mere anomalies and freaks. For instance, the tri-cuspid upper molar, as sometimes seen, especially in the Latin races of man in Europe, is a distinctly Lemurine character, as pointed out by Prof. E. D. Cope some time since. The special connection between the Lemurs and these races is only collateral, of course, and is as unknown as the paleontological history of man itself. But the knowledge of the Lemurine dental characteristics points out that the peculiarity is a reversion, according to the law of atavism, and that a scientific reason can be given to account for it. Or again, the wrinkled face of the lower wisdom tooth which appears in some persons, is a reappearance of the orang type, which has such molars. Or this tooth may be tri-cuspid as in the Macaiques. Again the vertical grooves on the face, and the notches on the edges of the incisors, which are so conspicuous in childhood when the incisors are first erupted, are rudiments of a permanent condition in some lower animals. The Galeopithecus monkey has the lower incisors divided into teeth like a comb, and many of the carnivora have the teeth deeply notched. In man the grooves and notches, which soon wear away, are suggestions indicating collateral inheritance, which in other forms is carried further and becomes permanent. Again, the canines are much reduced in size in man but still retain the backward curve that recalls the saber shape so conspicuous in the long trenchant canines of the carnivorous animals, and more or less so in the quadrumana. There is also a large diastema found in the apes and most monkeys, and before them in the carnivora, in front of the upper canine, between it and the lateral incisor, which the long lower canine enters when the lower jaw is closed. This diastema is sometimes found in the lower races of man, and very rarely there is a space between the upper canine and lateral incisor in civilized man, in well formed jaws (when it is not due to disease), which must be considered a survival. Again, the cusps of the grinding teeth of the insectivora are usually long and sharp for the crushing of the chittinous armour of insects. This peculiarity sometimes reappears in the extra long molar cusps of man; especially of the posterio-lingual cusp of the upper molars, which is often troublesome in articulating. The reappearance of extra cusps and cingulums on the human molars might, perhaps, be traced to the same source, as the molars of the Insectivora. Tomes says, "fairly bristle with cusps." Again, the New World monkeys have 36 teeth in very nearly all species, while the Old World quadrumana nearly all have the human formula, thirty-two, man being descended as a collateral branch with them. The higher ages have the human formula exactly, and also have the oblique ridge so characteristic of the human upper molars. This ridge is not found in the lower quadrumana, and seems to have been a later appearance.

Many other features in the teeth of man and their environments have been noticed which are explained by comparison with lower forms, and many more can be explained by further study and comparison. In fact, nearly all forms and features of the teeth, as of other organs, being inherited, come from previous forms and indicate relationship with them. And thus in our own branch we insist that the value of comparative anatomy cannot be over-estimated. It should be given a more prominent place in the curricula of our colleges, and in our text books, that the practitioner may gain a more scientific foundation for the principals that underlie practice. In this age of the world knowledge is of no value whatever if it is not exact to the very minu, tæ. In former times general knowledge was quite sufficient for the purposes of life, even if it was not very exact as to details; but in our day such general knowledge is not sufficient. Sometimes stupendous consequences depend upon the mastery of a minute detail. No fact is so insignificant as to be valueless, therefore it is worth all the labor that can be bestowed upon it to make it exact. In modern science much depends, we might say everything depends, upon exact knowledge of often apparently insignificant facts; but these, when marshalled in numbers give birth to great principles which revolutionize thought and affect the destinies of men.

